# Does 3D back contour changes following spinal fusion in children with idiopathic scoliosis?

**DOI:** 10.1186/1748-7161-9-S1-O61

**Published:** 2014-12-04

**Authors:** John Thometz, Xuecheng Liu, Channing Tassone

**Affiliations:** 1 Department of Orthopaedic Surgery, Medical College of Wisconsin, Milwaukee, WI, USA

## Background

Oftentimes, patients find trunk asymmetry, including thoracic rib hump, shoulder level difference, more troubling than a significant Cobb angle, as topographical deformity can be readily visualized. Evaluating improvement in these back contour parameters lends to additional criteria by which to assess post-surgical improvement. Quantec raster stereography is recognized as an accurate and reliable tool for monitoring three-dimensional back contour in patients with idiopathic scoliosis before and after surgery.

## Aims

To investigate the 3D back contour changes in adolescent idiopathic scoliotic deformity from surgical intervention using Quantec imaging.

## Design

In this prospective controlled study, 35 patients undergoing anterior and/or posterior spinal fusion were evaluated pre and post-operatively by Quantec Spinal Imaging System and radiography.

## Methods

Mean age of patients 14.5 years, with mean follow-up duration 1.7 years. A reliable Quantec protocol was established previously in the literature, consisting of twelve parameters. Pre and post-operative parameters were analyzed by paired t-test evaluating the effects of spinal fusion on scoliosis deformity.

## Results

Significant improvement was seen in thoracic Cobb angle from 52.9 to 19.6 degrees (p<.0001) and thoracolumbar from 54.7 to 21.3 degrees (p<.0001). This correlated with Quantec thoracic Q-angle improvement from 34.8 to 10.6 (p<.0001), and thoracolumbar Q-angle from 36.3 to 12.4 (p<.0001). Thoracic rotation improved from -8.6 to -5.6 (p=.042). Suzuki rib hump sum decreased from 15.7 to 11.5 (p=.016), and trunk asymmetry decreased from 40.6 to 24.1 (p<.0001). Left/right percent surface area also improved.

## Conclusions

This study builds upon previous evidence that spinal fusion produces measurable improvements in lateral curvature, trunk rotation, and topography deformity.

